# Psychological factors at work and musculoskeletal disorders: a one year prospective study

**DOI:** 10.1007/s00296-013-2843-8

**Published:** 2013-08-11

**Authors:** Joanna Bugajska, Dorota Żołnierczyk-Zreda, Anna Jędryka-Góral, Robert Gasik, Katarzyna Hildt-Ciupińska, Marzena Malińska, Sylwia Bedyńska

**Affiliations:** 1 Central Institute for Labour Protection–National Research Institute (CIOP-PIB), Czerniakowska 16, 00-701 Warszawa, Poland; 2 Institute of Rheumatology, Warszawa, Poland; 3 Warsaw School of Social Sciences and Humanities (SWPS), Warszawa, Poland

**Keywords:** MSDs, Psychosocial factors, Work demands

## Abstract

The etiology of musculoskeletal disorders is complex, with physical and psychosocial working conditions playing an important role. This study aimed to determine the relationship between psychosocial work conditions, such as psychological job demands, decision latitude, social support and job insecurity and musculoskeletal complains (MSCs) and (repetitive strain injuries (RSIs) in a 1-year prospective study. The job content questionnaire, the Nordic musculoskeletal questionnaire and provocation tests were used to study 725 employees aged 20–70 years. Pain in the lower back (58 % of subjects), neck (57 %), wrists/hands (47 %) and upper back (44 %) was most frequent. The carpal tunnel syndrome (CTS) (33.6 %), rotator cuff tendinitis (15.4 %), Guyon’s canal syndrome (13.4 %), lateral epicondylitis (7.6 %), medial epicondylitis (5.3 %), tendinitis of forearm–wrist extensors (7.8 %) and tendinitis of forearm–wrist flexors (7.3 %) were the most frequent RSIs. Logistic analysis showed that increased psychological job demands statistically significantly increased the probability of lateral and medial epicondylitis, and increased control (decision latitude) statistically significantly decreased the risk of CTS. There was no relationship between job insecurity, social support and the studied RSIs. Psychosocial factors at work predict prevalence of MSCs and RSIs, irrespectively of demographic factors, e.g., age or gender, and organizational and physical factors.

## Introduction

Maintaining work ability as long as possible and simultaneously the best health-related quality of life becomes a self-evident expectance of the society at large. Among occupationally active people, irrespective of their job type, some health problems occur more and more often and this tendency increases. Symptomatology of these problems might be different with repetitive strain injuries (RSIs) being one of the most common. Musculoskeletal complaints (MSCs), i.e., mainly regional pain in the locomotor system, also are often observed in that population. This means employees more and more frequently seek specialist rheumatic care.

The large body of literature focuses on MSDs, the synonym for RSIs, formerly also recognized under names of overload syndromes or overload injuries. The etiology of MSDs is complex, with occupational factors among the several causes conducive to the onset of those disorders. Physical factors related to the working environment and the way work is done such as body posture, forces exerted on the musculoskeletal system, repetitive movements, manual material handling, vibrations and microclimate are among the occupational factors that increase the risk of MSDs. Those factors are compounded by personal factors related to health and life style.

Since the beginning of the 1990s, there has been increased interest in the role of occupational psychosocial factors in the onset of MSDs [1–4]. Researchers have so far focused on various groups of workers and occupational factors, and most frequently they referred to those highlighted by Karasek [5, 6] in his model of work-related stress, such as psychological demands and decision latitude, than extended by social support. According to this model, high psychological demands, low decision latitude and low social support are related to stress and diseases. However, the findings of the studies exploring the link between these psychosocial factors and MSDs are inconclusive, and most of them have focused on a limited body regions, such as neck and shoulder [1, 7–10] or low-back pain [2, 11, 12].

Various pathomechanisms probably play a role in the *aetiopathogenesis* of MSCs. According to one theory, adverse psychosocial work factors increase physical load: high job demands result in increased exposure to effort over long work hours, few breaks at work and infrequent changes in posture. Van den Heuevel et al.’s [13] study supports this interpretation; it showed that the impact of psychosocial factors on the onset of complaints in the upper limb decreases with a decrease in physical work load.

Musculoskeletal pain can also develop irrespective of pathophysiological processes accompanied by tissue damage when (vulnerable or oversensitive) workers have such a perception of situations at work that they become aware of complaints that in less demanding conditions would have remained unnoticed [7, 14].

In addition to psychosocial factors, personal traits [15, 16] and coincidence of psychosocial factors and the demands of family life can also result in stress and an onset of MSCs.

This study aimed to determine the relationship between psychosocial factors at work, such as decision latitude, psychological job demands, social support and job insecurity, and the onset of MSCs and repetitive strain injury (RSI) in employees performing mental and physical work.

## Materials and methods

### Organization of the study

This was a prospective study. Its main principle consisted in examining twice, 12 months apart, the same group of employees. Employees reporting for periodic medical examinations participated in the study. Physicians, specialists in orthopedics, neurology and occupational medicine, who were familiar with the procedure of a physical examination of the musculoskeletal system according to the criteria document for evaluating the work-relatedness of upper-extremity musculoskeletal disorders [17], did the measurements. Participation in the study was voluntary. The Commission for Ethics in Scientific Studies at the Central Institute for Labour Protection–National Research Institute (CIOP-PIB) approved the protocol and the methods of the study. The study was performed in the different medium and large enterprises, in health, communications and industry sectors.

### Methods

The job content questionnaire [5] was used to diagnose psychosocial working conditions. It describes physical and psychological working conditions. The study covered the most frequently analyzed work characteristics such as job demands, decision latitude, social support and job insecurity.

The Nordic musculoskeletal questionnaire [17] studied MSCs in nine regions of the body (the neck, shoulders, lower back, upper back, elbows, wrists/hands, hips/thighs, knees and ankles/feet) in the past 12 months and in the past 7 days.

RSI was investigated with provocation tests followed in accordance with Sluiter et al. [18] protocol. The following disorders were diagnosed: radiating neck pain, Guyon’s canal syndrome, rotator cuff tendinitis, lateral epicondylitis, medial epicondylitis, carpal tunnel syndrome (CTS), tendinitis of forearm–wrist extensors and tendinitis of forearm–wrist flexors. The diagnosis of the syndrome was based on the positive results of at least one test.

### Statistical analysis

Statistical analysis was done with SPSS version 18.0. The following tests were used:

- Chi-squared test to compare the prevalence of individual RSIs in measurements I and II and the prevalence of pain-related complaints in measurements I and II;
- hierarchic logistic regression analysis to predict prevalence of RSIs and pain-related complaints in individual regions of the body on the basis of variables defining psychosocial factors of the working environment. Variables obtained in measurements I and II were the dependent variables. Predicting variables obtained in measurement II made it possible to a greater degree to draw conclusions on the cause and effect. The dependent variables in the logistic analysis were as follows:
  - complaints in nine regions of the body in the past 12 months,
  - at least one of the above mentioned RSIs.

Individual variables (age and gender), organizational and physical factors (working hours, repetitive work, force), were controlled in all analyses.

## Results

### Subjects’ characteristics

There were 725 employees in measurement I and 542 (74.8 %) in measurement II. The remaining persons did not take part in measurement II for unrelated reasons (dismissal, change in the type of work, long sick leave, pregnancy, etc.). In both measurements, females were in majority (77 %). Range–age workers participated in study were 20–70 years. We have not observed statistically significant differences in age between women and men. Table 1 lists their demographic and anthropometric characteristics.

**Table 1:** Subjects’ characteristics

Parameter	Total (SD)	Women (SD)	Men (SD)
Age (years)			
Measurement I	42.8 (9.9)	42.5 (9.9)	43.3 (10.3)
Measurement II	43.6 (10.1)	43.6 (9.8)	44.3 (10.3)
Tenure on the job (years)			
Measurement I	15.3 (11.2)	14.5 (10.8)	17.6 (12.1)
Measurement II	16.1 (11.2)	15.5 (10.9)	17.9 (11.9)
Total tenure (years)			
Measurement I	20.9 (10.5)	20.1 (10.2)	23.0 (11.1)
Measurement II	21.8 (10.6)	21.7 (10.3)	23.8 (11.2)
Body mass (kg)			
Measurement I	71.9 (14.3)	67.5 (12.4)	84.6 (11.7)
Measurement II	71.2 (14.1)	67.3 (12.1)	85.5 (11.7)
Body height (cm)			
Measurement I	167.6 (8.4)	164.1 (5.8)	177.5 (6.4)
Measurement II	167.2 (8.2)	164.2 (5.8)	177.9 (6.3)
Body mass index			
Measurement I	25.5 (4.2)	25.1 (4.3)	26.9 (3.7)
Measurement II	25.4 (4.1)	24.9 (4.1)	26.9 (3.6)

The employees had various jobs: work that was mostly mental (office workers), work that was mostly physical (toolmakers, welders, seamstresses, TV assembly workers, workers assembling electric elements and packers in the cosmetic industry) and work that was a combination of both (drivers, driving instructors and nurses). In measurement I, 29.1 % of the employees did mental work, 39.3 % did physical work and 30.6 % of employees did a combination of both. In measurement II, 33.2 % did mental work, 37.1 % did physical work and 29.7 % a combination of both.

#### MSCs

**Fig. 1:**
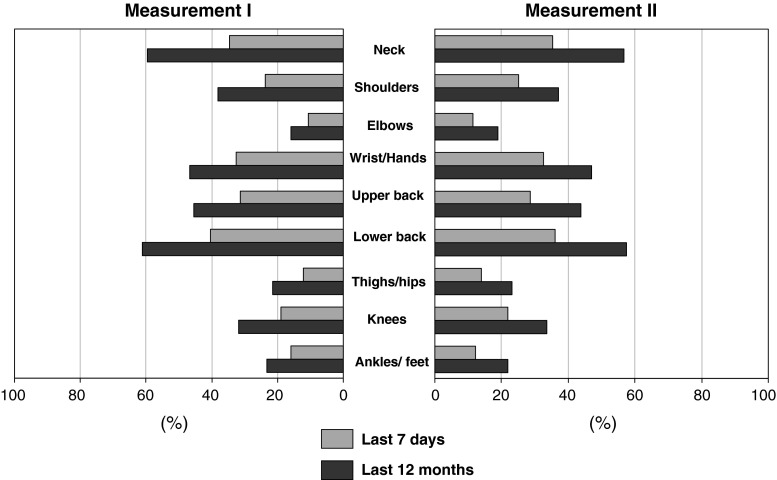
Comparison of the prevalence of musculoskeletal complaints in 2 measurements

The number of employees reporting in measurement II complaints in the neck, shoulders, upper back, lower back and ankles/feet both in the past 7 days and in the past 12 months was slightly lower than in measurement I. However, the number of subjects reporting in measurement II complaints in the elbows, wrists/hands, thighs and knees was slightly higher than in measurement I. Those differences were not statistically significant (Fig. 1).

#### RSI

Carpal tunnel syndrome was the most frequently diagnosed RSI in both measurements. The prevalence of all syndromes was lower in measurement II than in measurement I (Table 2). Those differences were statistically significant.

**Table 2:** The prevalence of repetitive strain injuries in 2 measurements

Parameter	Total (%)	Women (%)	Men (%)	*p*
Rotator cuff tendinitis				
Measurement I	22.5	23.4	19.8	0.52
Measurement II	15.4	18.6	6.0	0.001
Lateral epicondylitis				
Measurement I	12.2	15.0	4.3	0.002
Measurement II	7.6	9.0	3.4	0.05
Medial epicondylitis				
Measurement I	7.2	8.7	5.2	0.31
Measurement II	5.3	6.0	3.4	0.34
Carpal tunnel syndrome				
Measurement I	49.2	55.0	32.8	0.000
Measurement II	33.6	81.5	24.1	0.01
Guyon’s canal syndrome				
Measurement I	24.1	26.4	17.2	0.06
Measurement II	13.4	12.9	14.7	0.63
Tendinitis of forearm–wrist extensors				
Measurement I	8.9	9.9	6.0	0.26
Measurement II	7.8	9.0	4.3	0.11
Tendinitis of forearm–wrist flexors				
Measurement I	11.1	11.4	10.3	0.86
Measurement II	7.3	7.8	6.0	0.68

#### Predictors

Age and gender turned out to be the strongest predictors of RSI. With age, the probability of the onset of three RSIs statistically significantly increased: lateral epicondylitis by 8 % per year, medial epicondylitis by 11 % and tendinitis of forearm–wrist extensor by 5 % per year. In men, there was a statistically significantly lower probability of the onset of three RSIs: rotator cuff tendinitis by over 67 % per year, lateral epicondylitis by 72 % and CTS by 41 % per year. Mental work demands turned out to be a significant predictor of three RSIs, lateral epicondylitis, medial epicondylitis and tendinitis of forearm–wrist flexors and decision latitude—of CTS (Table 3). Age and gender were also strong predictors of the onset of MSCs, both in the past 7 days and in the past 12 months (Tables 4, 5).

**Table 3:** Relationship between psychosocial factors and selected repetitive strain injuries (hierarchic logistic regression analysis)

Independent variable	Repetitive strain injury						
	Rotator cuff tendinitis	Lateral epicondylitis	Medial epicondylitis	Carpal tunnel syndrome	Guyon’s canal syndrome	Tendinitis of forearm–wrist extensors	Tendinitis of forearm–wrist flexors
Age							
OR	1.025	1.084	1.106	0.988	0.978	1.050	1.019
*p*	0.103	0.001	0.001	0.318	0.156	0.024	0.371
95 % CI	0.995–1.056	1.035–1.136	1.043–1.172	0.966–1.011	0.948–1.009	1.007–1.095	0.978–1.061
Gender							
OR	0.335	0.282	0.542	0.588	1.151	0.461	0.758
*p*	0.011	0.050	0.357	0.052	0.684	0.173	0.577
95 % CI	0.144–0.777	0.079–0.998	0.148–1.994	0.344–1.004	0.585–2.263	0.152–1.403	0.287–2.005
Decision latitude							
OR	0.986	1.015	0.978	0.974	1.025	0.983	1.010
*p*	0.386	0.537	0.417	0.037	0.167	0.451	0.666
95 % CI	0.954–1.018	0.968–1.065	0.926–1.033	0.950–0.998	0.990–1.060	0.940–1.028	0.965–1.057
Mental job demands							
OR	1.050	1.102	1.103	1.001	0.996	1.073	1.103
*p*	0.092	0.017	0.038	0.961	0.893	0.069	0.013
95 % CI	0.992–1.110	1.017–1.194	1.005–1.209	0.957–1.047	0.935–1.060	0.995–1.158	1.021–1.192
Job insecurity							
OR	1.122	0.922	0.963	0.988	1.183	1.194	1.215
*p*	0.233	0.595	0.828	0.873	0.095	0.173	0.129
95 % CI	0.929–1.355	0.685–1.242	0.687–1.350	0.847–1.151	0.971–1.442	0.925–1.541	0.945–1.564
Social support							
OR	1.003	1.053	1.084	1.038	1.003	1.121	1.060
*p*	0.952	0.441	0.304	0.341	0.957	0.101	0.395
95 % CI	0.911–1.104	0.923–1.203	0.929–1.266	0.962–1.120	0.903–1.114	0.978–1.285	0.927–1.212
Physical job demands							
OR	1.232	1.007	0.744	1.307	0.735	0.893	0.837
*p*	0.211	0.974	0.268	0.036	0.074	0.595	0.427
95 % CI	0.889–1.707	0.666–1.523	0.441–1.255	1.018–1.677	0.524–1.030	0.588–1.356	0.540–1.298

**Table 4:** Relationship between musculoskeletal complaints experienced in the past 7 days and selected psychosocial factors (hierarchic logistic regression analysis)

Independent variable	Subjective complaints in the past 7 days								
	Neck	Shoulders	Elbows	Wrists/hands	Upper back	Lower back	One/both thighs/hips	One/both knees	One/both ankles/feet
Age									
OR	1.00	1.02	1.04	1.01	0.99	1.01	1.05	1.03	1.05
*p*	0.58	0.14	0.03	0.20	0.43	0.45	0.00	0.02	0.01
95 % CI	0.98–1.03	0.99–1.04	1.00–1.08	0.99–1.04	0.96–1.01	0.98–1.03	1.02–1.09	1.00–1.06	1.01–1.09
Gender									
OR	0.45	0.71	1.30	0.84	0.48	0.82	0.76	1.22	1.43
*p*	0.00	0.28	0.51	0.54	0.02	0.45	0.48	0.50	0.35
95 % CI	0.26–0.80	0.39–1.31	0.59–2.84	0.47–1.48	0.26–0.88	0.49–1.38	0.36–1.61	0.69–2.16	0.67–3.07
Decision latitude									
OR	1.00	0.97	0.97	0.96	0.99	0.99	0.98	1.00	0.98
*p*	0.72	0.08	0.21	0.00	0.63	0.62	0.30	0.99	0.28
95 % CI	0.98–1.03	0.95–1.00	0.94–1.01	0.93–0.98	0.96–1.02	0.97–1.02	0.95–1.01	0.97–1.03	0.94–1.02
Mental job demands									
OR	1.02	1.07	1.08	1.11	1.04	0.98	1.00	1.03	1.10
*p*	0.35	0.00	0.02	0.00	0.10	0.43	0.92	0.19	0.00
95 % CI	0.97–1.07	1.02–1.13	1.01–1.16	1.06–1.17	0.99–1.09	0.94–1.03	0.94–1.06	0.98–1.09	1.03–1.17
Job insecurity									
OR	0.99	1.11	1.24	1.18	1.02	0.97	0.98	0.95	1.04
*p*	0.87	0.21	0.45	0.03	0.75	0.74	0.88	0.57	0.74
95 % CI	0.85–1.15	0.94–1.30	1.00–1.53	1.01–1.38	0.87–1.20	0.84–1.13	0.80–1.21	0.80–1.13	0.84–1.29
Social support									
OR	0.97	1.06	1.09	1.08	0.97	0.95	0.93	0.99	0.98
*p*	0.40	0.16	0.15	0.06	0.44	0.18	0.20	0.76	0.71
95 % CI	0.89–1.04	0.97–1.15	0.97–1.22	0.99–1.17	0.89–1.05	0.88–1.02	0.84–1.03	0.90–1.07	0.87–1.09
Physical job demands									
OR	0.98	1.29	1.46	1.56	1.36	1.12	0.86	0.99	1.44
*p*	0.92	0.07	0.05	0.00	0.02	0.34	0.38	0.93	0.05
95 % CI	0.77–1.27	0.98–1.69	0.99–2.13	1.20–2.02	1.04–1.77	0.88–1.44	0.61–1.21	0.74–1.31	1.00–2.06

**Table 5:** Relationship between limitations in activity related to complaints in the past 12 months and selected psychosocial factors (hierarchic logistic regression analysis)

Independent variable	Subjective complaints in the past 12 months								
	Neck	Shoulders	Elbows	Wrists/hands	Upper back	Lower back	One/both thighs/hips	One/both knees	One/both ankles/feet
Age									
OR	1.03	1.02	1.05	1.01	1.03	1.03	1.05	1.04	1.03
*p*	0.04	0.12	0.02	0.38	0.06	0.03	0.01	0.01	0.07
95 % CI	1.00–1.05	0.99–1.05	1.01–1.09	0.98–1.04	1.00–1.05	1.00–1.05	1.01–1.08	1.01–1.07	1.00–1.07
Gender									
OR	0.33	0.46	1.08	0.43	0.56	0.78	0.38	0.88	1.36
*p*	0.00	0.05	0.86	0.02	0.10	0.40	0.05	0.70	0.40
95 % CI	0.16–0.67	0.21–1.01	0.45–2.60	0.21–0.88	0.28–1.13	0.45–1.38	0.14–1.02	0.46–1.69	0.65–2.84
Decision latitude									
OR	1.00	0.98	0.98	0.98	0.96	0.98	0.98	0.98	0.98
*p*	0.88	0.23	0.52	0.14	0.01	0.24	0.30	0.29	0.34
95 % CI	0.97–1.03	0.95–1.01	0.94–1.03	0.95–1.01	0.93–0.99	0.96–1.01	0.94–1.02	0.95–1.01	0.95–1.02
Mental job demands									
OR	1.00	1.04	1.07	1.08	1.03	1.02	1.03	1.05	1.08
*p*	0.91	0.12	0.07	0.00	0.27	0.41	0.29	0.08	0.01
95 % CI	0.97–1.03	1.00–1.10	0.99–1.15	1.03–1.14	0.98–1.09	0.97–1.07	0.97–1.10	0.99–1.11	1.02–1.16
Job insecurity									
OR	0.94	0.14	1.01	1.08	0.97	0.97	1.05	0.77	1.00
*p*	0.50	0.15	0.92	0.38	0.78	0.70	0.67	0.01	0.95
95 % CI	0.79–1.12	0.95–1.37	0.79–1.29	0.91–1.28	0.82–1.16	0.83–1.36	0.84–1.30	0.63–0.95	0.82–1.23
Social support									
OR	0.90	1.02	0.99	0.95	0.96	0.97	0.93	0.97	0.87
*p*	0.02	0.70	0.90	0.25	0.47	0.48	0.22	0.49	0.01
95 % CI	0.82–0.99	0.92–1.12	0.87–1.13	0.86–1.04	0.88–1.06	0.89–1.05	0.83–1.04	0.88–1.06	0.77–0.97
Physical job demands									
OR	1.23	1.21	1.11	1.57	1.40	1.03	1.07	0.87	1.15
*p*	0.14	0.23	0.61	0.00	0.02	0.84	0.70	0.38	0.41
95 % CI	0.93–1.64	0.88–1.66	0.74–1.68	1.18–2.09	1.04–1.87	0.79–1.33	0.75–1.53	0.65–1.18	0.82–1.61

Psychological job demands have also been found to be a significant predictor of pain in the past 7 days in the shoulders, elbows, wrists/hands and ankles/feet; decision latitude and pain in the wrists/hands; and job insecurity was predictor of pain in the knees (Table 4). On the other hand, for complaints in the past 12 months, psychological job demands significantly predict pain in the shoulders; decision latitude predicts pain in the upper back; job insecurity significantly predicts pain in the knees; and social support predicts pain in the neck and ankles/feet (Table 5).

## Discussion

The problem of the prevalence of musculoskeletal pain (MSCs) and RSI, which the working environment can cause, is rarely discussed in the literature on rheumatology. However, this is an increasing clinical problem, and thus, rheumatologists should be made aware of it.

Studies published over 10 years old estimated that MSCs were prevalent in ~15 % of workers [19, 20]. In a later study, with 869 workers from various occupational groups (manual handlers, delivery drivers, technicians, customer services, computer operators and general office staff), the prevalence of complaints was higher. Thirty-four percent of subjects reported pain-related complaints in the neck, 35 % in the shoulders, 17 % in the elbows and 35 % in the wrists/hands [21]. According to Walker-Bone et al.’s [22] questionnaire survey, almost 20 % of 6,055 persons complained of pain in the neck and in the upper limbs. According to Roquelaure et al. [23], over 50 % of 2,685 subjects reported MSCs. Pain-related complaints are also common in computer operators. According to Sillanpaa et al. [24], 63 % of them reported pain in the neck, 24 % in the shoulders, 18 % in the elbows, 35 % in the forearms and 16 % in the back. According to Bugajska et al.’s [25] Polish study 14–64 % of women and 13–55 % of men performing repetitive tasks reported pain in different parts of the body. Women most frequently reported complaints in all the regions of the body that were studied. Lam and Thurstone [26] and de Zwart et al. [27] confirmed those results. Our study’s results showed that the most common complaints were those in the lower back (58 % of subjects), the neck (57 %), the wrists/hands (47 %) and the upper back (44 %).

It is interesting that in measurement II, after 12 months, there were generally fewer complaints of pain in individual regions of the body; there were fewer RSIs, too. The time factor usually plays a negative role in rheumatic disorders: the patient’s condition deteriorates with time. In this case, the reverse was true. It is possible that workers whose health significantly deteriorated did not participate in measurement II. This is the so-called healthy worker effect.

MSCs are often temporary, and they are a reaction to short-term excessive musculoskeletal load. They disappear once the effort stops, so their prevalence in workers was higher than the prevalence of RSIs. According to Roquelaure et al. [23], over 50 % of 2,685 employees reported complaints of nonspecific MSCs, whereas 13 % of them were clinically diagnosed with at least one RSI. Similarly, in Walker-Bone et al.’s [2] study, only ~20 % of persons with musculoskeletal pain had clinical bases for diagnosing one of the 11 defined RSIs.

In the present study, too, RSIs diagnosed on the basis of provocation tests were less frequent than pain-related complaints. CTS, diagnosed in 33.6 % of the employees, was the most common one, followed by rotator cuff tendinitis in 15.4 %, Guyon’s canal syndrome in 13.4 %, lateral epicondylitis in 7.6 %, medial epicondylitis in 5.3 %, tendonitis of forearm–wrist extensors in 7.8 % and tendinitis of forearm–wrist flexors in 7.3 % of the subjects. We also observed statistically significant differences between women and men in prevalence of CTS and lateral epicondylitis, consequently occurred in both measurements. This finding is in line with above mentioned outcome showing that women more frequently report the musculoskeletal complains.

We are aware that the number of occurrences of MSC and RSI in our studies is high; it would certainly be lower if we had confirmed this using objective diagnostic methods. About 70 % of worker in our study performed work that was mostly physical (toolmakers, welders, seamstresses, TV assembly workers, workers assembling electric elements and packers in the cosmetic industry) and work that was a combination of both (drivers, driving instructors and nurses). In the above mentioned occupations, an increased risk of MSD might occur. Nevertheless, the results represent an actual problem faced in the occupational environment, and we claim that each case of employee musculoskeletal complaint needs observation and often modification of work performance and, possibly, even specialist consultation and treatment.

An increase in those complaints in employees performing mental work inspired researchers to look into the working environment for causes of MSCs other than physical factors. They focused on the psychosocial factors at work which, independently or in an interaction with physical factors, could be the cause. In our study, we also found the significant impact of physical factors on prevalence on MSDs (wrist/hands and upper back) and RSI (Carpal Tunnel Syndrome). Therefore, the combined effect of psychosocial and physical factors is also likely to occur and should be considered in the further analysis of our data.

Earlier literature on the subject showed a positive relationship between work-related stress and prevalence of MSDs, especially in the neck [9]. Most studies pointed to high psychological job demands as a source of psychosocial stress in people with those complaints. There is also proof that low decision latitude, understood as workers’ influence on their work, is also responsible for pain-related complaints in the upper section of the spine. According to critics of those studies, because they are cross-sectional and not longitudinal, it is impossible to unequivocally state that those psychosocial job conditions cause MSCs. Moreover, conclusions from those studies are difficult to generalize because of the varied ways of conceptualizing psychosocial job characteristics and the tools they were measured with.

This study aimed to avoid the methodological weaknesses of previous studies. Firstly, it was longitudinal, and it was conducted twice, one year apart, each time considering symptoms in the past 12 months and in the past 7 days. The methodology of this study increases the power of the cause-and-effect predictions. Secondly, the research assumptions were based on Karasek’s conception, which is well established in the psychology of stress; it points to three basic dimensions of stress: psychological job demands, decision latitude and social support [27]. The study also considered the authors’ latest modification of this conception, which consists in introducing another important source of stress at work into the model, i.e., job insecurity. In previous studies, on the relationship between psychosocial work characteristics and MSCs, not enough attention was devoted to this currently common threat. In the present study, job insecurity turned out to be a significant positive predictor of complaints related to the region of the elbows and wrists reported in the past 12 months and in the past 7 days. The results confirm the correctness of considering that psychosocial variable in predicting MSCs.

This study also proved that the other classic dimensions of Karasek’s model really predict the prevalence of MSCs. Psychological job demands turned out to be the strongest one. They caused a significant increase in complaints in the elbows and wrists, and ankles/feet in the past 12 months and in the elbows, wrists, ankles/feet and shoulders in the past 7 days. The importance of psychological job demands in the *pathogenesis of* MSDs has also been confirmed by the results that show that they significantly contribute to the development of lateral epicondylitis and medial epicondylitis. The present results related to psychological job demands thus confirm the results of other studies [12, 13].

This study also showed low decision latitude as another predictor of MSCs; the lower the decision latitude, the stronger the short- and long-term complaints in the wrists/hands, i.e., in the past 7 days and in the past 12 months. Moreover, low job decision latitude coincided with the prevalence of CTS. It should be pointed out that the present results are among the first in the literature that prove that long-term low decision latitude, understood as workers’ lack of influence over the pace of work and breaks at work, is important for the occurrence of MSCs and their developing into RSIs. This finding is in step with other studies [12, 27–32]. Social support turned out to be the weakest predictor of the complaints in the current study. Low social support at work caused an increase in complaints of pain in the region of the neck only. This is so probably because the effect of this occupational stressor is very nonspecific.

All individual (e.g., age and gender), organizational and physical variables (working hours, repetitive work, force), which are considered as inherent risk factors, were controlled in the study, and therefore, the influence of psychosocial factors on MSC’s and RSI was not contaminated by these variables.

Other researchers point to other changes that can affect the prevalence of MSCs such as style of work [33], excessive involvement in work [34] and individual abilities to cope with stress at work. Considering those parameters in future analyses would provide a promising confirmation of the recently cited in literature model of a relationship between psychosocial factors and MSCs called the “Cinderella model,” which assumes that some psychological features, such as perfectionism, can lead to overuse of low-threshold motor units in muscles [35, 36]. According to this concept, personal characteristics cause significant job demands to coincide with other demands of personal life (e.g., family ones) and thus make a good life style impossible (e.g., lack of time and motivation for physical exercise) and together they result in stress and an onset of musculoskeletal disorders. The results presented in this paper are a stimulus to conduct such holistic analyses.

In summary, psychosocial factors are a positive predictor of the prevalence of MSCs and RSIs, irrespective of personal factors (age or gender), organizational and physical factors (working hours, repetitive work, force). Thus, those factors should not be neglected by rheumatologists during their routine practice. In an analysis of the etiology of the aforementioned health problems and it should be borne in mind that apart from activity in the private life, adverse psychosocial factors at work also play a role and increase the work-related physical load. The role of prevention of MSDs at the organizational level in the workplace and pro-health behaviors of the workers themselves should not be underestimated.
